# Effective DNA Inhibitors of Cathepsin G by *In Vitro* Selection

**DOI:** 10.3390/ijms9061008

**Published:** 2008-06-20

**Authors:** Barbara Gatto, Elena Vianini, Lorena Lucatello, Claudia Sissi, Danilo Moltrasio, Rodolfo Pescador, Roberto Porta, Manlio Palumbo

**Affiliations:** 1 Department of Pharmaceutical Sciences, University of Padova, Via Marzolo 5, 35131 Padova, Italy; 2 Gentium S.p.A., Piazza XX Settembre 2, 22079 Villa Guardia, Italy

**Keywords:** Cathepsin G, defibrotide, Selex, TG repeats, alternating polynucleotides, CatG: Cathepsin G, Selex: Systematic evolution of ligands by exponential enrichment, PCR: polymerase chain reaction, ssDNA: single strand DNA; PAGE: Polyacrilamide gel electrophoresis, SPR: Surface Plasmon Resonance

## Abstract

Cathepsin G (CatG) is a chymotrypsin-like protease released upon degranulation of neutrophils. In several inflammatory and ischaemic diseases the impaired balance between CatG and its physiological inhibitors leads to tissue destruction and platelet aggregation. Inhibitors of CatG are suitable for the treatment of inflammatory diseases and procoagulant conditions. DNA released upon the death of neutrophils at injury sites binds CatG. Moreover, short DNA fragments are more inhibitory than genomic DNA. Defibrotide, a single stranded polydeoxyribonucleotide with antithrombotic effect is also a potent CatG inhibitor. Given the above experimental evidences we employed a selection protocol to assess whether DNA inhibition of CatG may be ascribed to specific sequences present in defibrotide DNA. A Selex protocol was applied to identify the single-stranded DNA sequences exhibiting the highest affinity for CatG, the diversity of a combinatorial pool of oligodeoxyribonucleotides being a good representation of the complexity found in defibrotide. Biophysical and biochemical studies confirmed that the selected sequences bind tightly to the target enzyme and also efficiently inhibit its catalytic activity. Sequence analysis carried out to unveil a motif responsible for CatG recognition showed a recurrence of alternating TG repeats in the selected CatG binders, adopting an extended conformation that grants maximal interaction with the highly charged protein surface. This unprecedented finding is validated by our results showing high affinity and inhibition of CatG by specific DNA sequences of variable length designed to maximally reduce pairing/folding interactions.

## 1. Introduction

Cathepsin G (CatG) is a serine protease commonly found in the azurophilic granules of polymorphonuclear leukocytes and mast cells [1]. Together with elastase and proteinase 3 it belongs to the chymotrypsin family and cleaves extracellular matrix proteins such as elastin, collagen, fibronectin and laminin causing extensive lung tissue damage [2].

As for other peptidases, several biological functions can be exerted by CatG. In blood clotting the enzyme is involved in an alternative pathway of leukocytes initiation of coagulation by activating coagulation factor X [3] and factor V [4]; it can also cleave and potentially modulate thrombin receptor and activate platelets *in vitro* [5]. CatG can degrade necrotic tissues and is therefore related to several pulmonary inflammatory diseases like lung emphysema [6], bronchitis [7] and cystic fibrosis [8], as well as a variety of other pathological conditions associated with inflammation [9].

The enzymatic activity of Cathepsin G is physiologically regulated by two types of proteinase inhibitors: the so called “canonical” inhibitors and the serpins (serine protease inhibitors). The former are relatively small proteins (29–190 amino acids) acting as tight-binding reversible inhibitors; among them Mucus Proteinase Inhibitor (MPI) [8], eglin c [10] and aprotinin deserve to be mentioned [11]. Serpins are larger proteins (400–450 residues) that form an irreversible complex with their target protein through the formation of a non-hydrolysable acyl bond between the catalytic site of the protease and their reactive site loop. Among serpins, α1-antichymotrypsin is the most relevant inhibitor of CatG. These kinds of inhibitors are not suitable for therapeutic use. In fact, they are all non selective as they can bind and inhibit other chymotripsins [12]. Besides, their stability and distribution in vivo is strongly affected by their peptidic nature. Thus, the research is mainly directed to the discovery and development of non-peptidic inhibitors of CatG with higher selectivity.

Negatively charged macromolecules are effective inhibitors of cathepsin G: this is not surprising, since the enzyme is a very basic protein, exhibiting 36 positively charged residues not compensated by neighbouring counter charges [13]. CatG is indeed able to form 1:1 complexes with low molecular weight heparins [14], as well as with heparin-like dextran derivatives and glycosamminoglycans [15–17]. However, heparins exhibit undesired side effects due to their low specificity for this target.

A polyanion macromolecule as DNA was shown to be able to bind and inhibit the enzyme *in vitro* and *in vivo* [18–20]. In particular, DNA fragments shorter than 0.5 kb of genomic origin like those produced by DNAse treatment in patients with cystic fibrosis [21] as well as a 30 bp DNA fragment bind CatG very efficiently at physiological conditions [20]. These short DNA fragments with random sequences have higher affinity for cathepsin G than for human neutrophil elastase and proteinase 3, specificity in accordance with the decreasing cationic character and different localization of arginines [21].

Short nucleic acid fragments could hence be considered as specific CatG inhibitors that, differently from serpins, act in a reversible way. In fact, defibrotide, a complex mix of single stranded polydeoxyribonucleic acid sequences of genomic origin with therapeutic use [22–26] is an effective inhibitor of cathepsin G, and its antithrombotic effect has been ascribed to the anti-cathepsin G activity [27]. We decided to investigate on the binding specificity of defibrotide to the enzyme, i.e. to understand whether the recognition of CatG is mediated by specific sequence patterns in the context of genomic DNA. In heparin-like dextran compounds recognition of the enzyme has been examined in detail, showing that specific derivatives are responsible for CatG regulation: ionic interactions guide the recognition with the positively charged protein, but the initial electrostatic complex is then stabilized by non-ionic binding to CatG [16].

We therefore asked if, in a similar manner, specific sequences present in defibrotide DNA would contribute to stabilize complexation with CatG. To identify these sequences, we applied a Selex protocol (Systematic evolution of ligands by exponential enrichment) [28] in order to identify ssDNA molecules with high affinity for Cathepsin G starting from a highly diverse random pool of synthetic oligodeoxyribonucleotides mimicking the natural defibrotide of genomic origin. Selex allows the screening and PCR amplification of the random-sequence pool followed by identification of the best binders to the desired target, and it is based on the assumption that some oligonucleotides in the highly diverse combinatorial pool may possess desirable features to assure selectivity to the target molecule. Although inhibition is not always demanded by the selection protocol, in many instances these ligands inhibit the biological functions of the targeted proteins as in pegaptinib, a pegylated aptamers against VEGF. It is remarkable that, although oligonucleotides stability in vivo is low, aptamers half life in biological fluids can be improved through pre- and post-Selex modifications: pegaptinib is in fact a marketed drug [29].

Indeed, our selection allowed us to characterize several single stranded oligodeoxyribonucleotides with optimal sequence and length that tightly bind Cathepsin G. The analysis of the selected CatG binders unveiled the presence of imperfect TG repeats as common motifs and allowed us to prove that alternating oligopolymers with extended conformations and precise length are preferred CatG binders and inhibitors.

## 2. Results

### 2.1 Selection of CatG binders from a random DNA pool

Specific Cathepsin G binders were selected from a synthetic oligodeoxyribonucleotide pool 96 nucleotides long; the randomised 60 central bases has a length and theoretical diversity mimicking the molecular diversity of defibrotide (average molecular weight ≅ 21 KDa); the random combinatorial region is flanked by two conserved regions of 18 nucleotides needed for PCR amplification and cloning steps.

CatG has a theoretical pI of 11.0, thus in our working conditions (pH 7.5) it shows a positive net charge. This property allowed us to incubate CatG and DNA in solution, and perform selection with a cation exchanger resin like sulphopropyl (SP) Sepharose. In this system, the non-specific binding of DNA molecules to the underivatized Sepharose SP resin is minimal and non-influential: only CatG, free or DNA bound, is retained onto the column, while those DNA sequences not efficiently bound to the enzyme can be easily washed away prior to elution.

**Figure 1.**
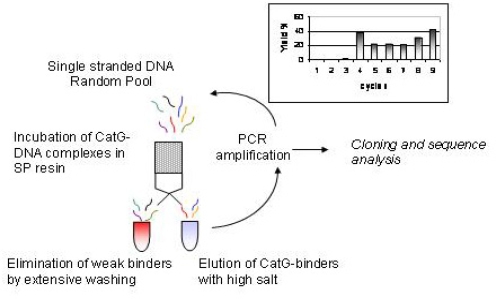
Flowchart of the selection of binders for Cathepsin G from a single stranded DNA combinatorial pool. Each selection cycle shown in the figure was reiterated until enrichment of the initial pool in CatG binders was obtained (final yield 42%). Yields at each cycle are shown in the inset. Precolums were applied between cycles 6 and 7 and 8 and 9 in order to avoid non-specific binding to the resin. Relative DNA/CatG concentrations and washing volumes were changed through the selection to modulate stringencies (see text).

The incubation between DNA and the protein was performed in the presence of NaCl, KCl and MgCl_2_ (150, 5 and 5 mM respectively). High salt concentration could favour selectivity in binding by reducing the expected strong electrostatic interactions between oligonucleotides and protein, as shown by Duranton [20]. Additionally, monovalent cations like sodium and potassium may stabilize G quartets, which are among the most common folding motifs exhibited by ssDNA aptamers [30]. The CatG-bound DNA molecules, labelled at their 5’ end with ^32^P, were efficiently removed from the column using a higher ionic strength buffer (EB buffer, 0.8 M NaCl), amplified by PCR and then reduced to single stranded molecules to be used for the reiteration of Selex cycles. A summary of the selection protocol and the yields obtained are reported in Figure 1.

The first four cycles of selection were conducted with relatively low immobilized protein (4–12 ng/ul). Not surprisingly, at cycle 4 a significant increase in the yield of DNA binders was observed, reflecting the favourable electrostatic binding. To reduce yield the concentration of immobilized protein was reduced and washings were increased until the final cycles, where higher protein concentration were allowed. Two precolumn cycles were used in order to avoid the minimal unspecific binding of the DNA to the matrix of the resin.

The Selex was terminated after the ninth cycles: at this point the enrichment of the pool, considering the final yield of 42 %, was considered satisfactory. To confirm this, three supplementary rounds of selection (not shown) did not produce further increase in pool affinity to CatG.

The selected oligodeoxyribonucleotide molecules were finally PCR amplified, cloned in *E.coli* and sequenced through Sanger method. 19 different sequences from positive clones are listed in Table 1.

### 2.2 Identification of structural motifs in DNA - CatG binding

The DNA sequences obtained by the selection and shown in Table 1 were analyzed with FastA ALIGN and Clustal X [31–33] to evidence if common motifs were present among the 20 different identified sequences of CatG binders. Alignment of sequences however did not evidence a common consensus. Hence, we analyzed the DNA with Mfold [34], a program able to evaluate the formation of stable folding raising from inter- or intra-molecular Watson Crick base pairing. Mfold analysis of CatG DNA binders yielded very low energy values associated to the predicted folded conformations of DNA molecules, with average free energy values around −3 kcal/mol. These findings support the observation that no common sequence can be found in the selected CatG binders, pointing out the lack of a precise three-dimensional motif related to the recognition of the protein.

None of the selected CatG binders tend to self-dimerize into double stranded conformations: a close examination of the sequences as well as their predicted folding evidenced long stretches of unpaired sequences characterized by short alternating dTdG or dGdT. Sequences have an average content of Ts and Gs around 65%. Among these, clones CG1, CG3, CG11, CG16, CG20, CG28, CG45, CG48, CG49 and CG51 were those enriched in TG elements.

Indeed, it is well known that eukaryotic genomes contain many short tandem repeats of very simple motifs (usually dinucleotides) [35], and the human genome has approximately 10^5^ copies of stretches of dT−dG alternating sequence [36]. TG elements are randomly dispersed in the genome but are stable components present throughout the evolution of eukaryotic genomes, with a molecular size between 20 and 60 dinucleotide repeats [37]. Since defibrotide is a single strand polydeoxyribonucleotide resulting from digestion of mammalian genomic DNA, it is likely that TG elements are highly represented motifs, possibly responsible for Cathepsin G recognition and inhibition. If this is the case, the selected clone sequences had identified alternating non-self complementary oligodeoxyribonucleotides of defined length as a class of CatG binders.

To prove this hypothesis we compared the affinities of several linear alternating oligodeoxyribonucleotides employing the protocol used for the Selex. The sequence CG51 has a predicted folding at 150 mM NaCl and 5 mM Mg^2+^ of −2.52 kcal/mol, well in the range of those exhibited by all other selected CatG binders, and it has therefore been chosen as representative of DNA-CatG binders. A quantitative analysis of the oligo-protein binding process allowed to compare the dissociation constant of CG51 with that of the linear alternating polymer (dT-dG)_30_. As controls, we measured the affinities of the alternating non self-complementary oligodeoxyribonucleotide (dA-dC)_30_ and of the complementary sequence of CG51, cCG51. Defibrotide, whose molecular weight is consistent with those of other oligos, represents our random sequence control. Additional controls are two aptamers identified in Selex protocols toward different targets. One is pE35, a 60 bases aptamer selected in our lab [38] with good affinity and specificity toward L-Tyr [39]. The second is a short (5 KDa) and highly structured oligo, THR, a DNA aptamer with G-quartets arrangement selected against the serine protease thrombin [40].

**Figure 2.**
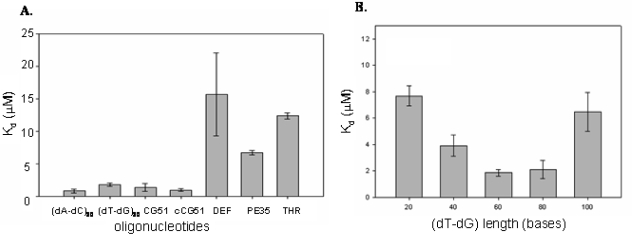
A) CatG binding properties of tested oligonucleotides. The dissociation constants (K_d_) of selected CatG binders, the TG oligonucleotides (dT-dG)_30_ and appropriate controls were evaluated by chromatography and reported as a function of DNA sequence. B) CatG binding properties of TG oligonucleotides. The dissociation constants (K_d_) of different TG oligonucleotides were analyzed as a function of DNA chain length.

As reported in Figure 2A, we found that the selected aptamer CG51, its complementary strand cCG51, as well as (dT-dG)_30_ and (dA-dC)_30_, showed comparable binding affinity values, proving our hypothesis that CatG Selex had helped identify linear alternating oligodeoxyribonucleotides rich in dinucleotide repeats as enzyme binding motifs. The folded G-quartet organized THR aptamer does not show an appreciable affinity for the protein, as well as pE35, an aptamer for a different target with comparable length to CG51. Defibrotide showed the lowest affinity for CatG among all the tested oligonucleotides, consistently with its sequence complexity.

Since TG repeats found in genome have a molecular size ranging from 20 to 60, we explored the effect of length of TG repeats in CatG recognition evaluating the dissociation constants of a number of (dT-dG)_n_ derivatives (where n is the degree of polymerization) (Figure 2B).

As summarized in Figure 2B, irrespective of the nucleotide sequence, we found a bell shaped dependence of K_d_ as a function of aptamer length, with molecules 60–80 nucleotides long being the best binders, consistently with the size of the oligonucleotides random pool employed in the Selex protocol as well as of defibrotide.

**Figure 3.**
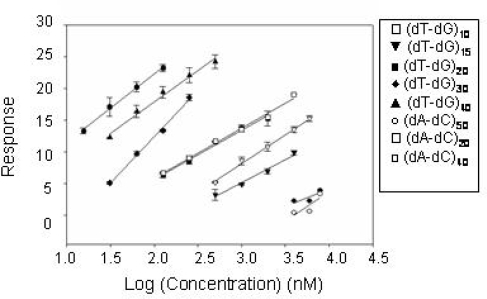
CatG binding properties of TG and AC oligonucleotide series. Dependence of CatG binding upon oligonucleotide concentration for (dT-dG)_n_ and (dA-dC)_n_ sequences determined by SPR data.

The CatG-DNA binding process was additionally monitored by SPR, a different technique employing immobilized CatG onto a chip surface. Several linear (dT-dG)_n_ and (dA-dC)_n_ oligodeoxyribonucleotides of different length were tested and their dissociation from enzyme compared. A first data set was generated using a variable molar concentration of each oligonucleotide. Results are summarized in Figure 3, where the Log concentration-effect curves using the test sequences, referring to the concentration range over which a linear regression was obtained, are reported.

Under these conditions, (dT-dG)_50_ appears to be a somewhat more potent binder than (dT-dG)_40_ , although the latter yields the largest resonance signal. CatG recognition is substantially poorer with 20–30mers. It should be noted that increasing the chain length over 60 bases brings forth an increase in binding affinity but this increment is less steep than that in the range 30–60. This rank order is shared by all the sequences tested although we found a reduced binding for (dA-dC)_n_ derivatives in comparison to (dT-dG)_n_ ones at the high molecular weight.

### 2.3 Inhibition of CatG catalytic activity

**Figure 4.**
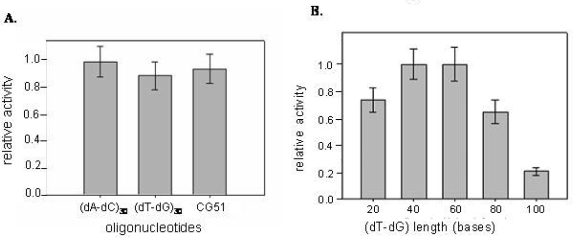
CatG inhibition properties of selected oligodeoxyribonucleotides. The relative inhibition data are reported as a function of DNA sequence (Panel A) or (dT-dG) chain length (Panel B).

The enzymatic activity of CatG was determined in the presence of increasing concentration of the selected CatG binders and of the alternating (dT-dG) repeats. The catalytic inhibition properties were evaluated by monitoring the reduction in the hydrolysis rate of an appropriate chromogenic peptide substrate. According to literature data, an hyperbolic non-competitive tight binding mechanism for the DNA-CatG interaction has been confirmed [20]. In fact DNA sequences inhibit enzyme activity at concentrations comparable to the protein (nM) concentration. The inhibitory effects obtained with DNA chains of different sequences but comparable length appear to be poorly affected by the sequence (Figure 4A). However, a role of the chain length clearly emerges using the TG repeats series, the inhibitory activity of which varies substantially with the number of residues (Figure 4B).

In particular, while chains 20–60 bases long share comparable activity, longer chains exhibit a progressive increment in their inhibitory efficiency. Additionally, a residual enzymatic activity of about 25% is retained by the protein-DNA complex when the oligonucleotide chain length does not exceed 40 bases, whereas it is almost doubled for oligos 60 or more bases long. This effect can be explained by the fact that the shorter oligonucleotides more likely interact with CatG on a 1:1 basis [16], whereas in the presence of longer sequences more than one protein molecule can bind simultaneously to each oligonucleotide chain. The inhibition effect of oligos on CatG was not affected by the presence of a charged protein like albumin, indicating that inhibition is selective (not shown).

## 3. Discussion

The *in vitro* selection of CatG binders was successfully accomplished and allowed to identify the oligodeoxyribonucleotide sequences that bind tighter to the enzyme within a highly complex population of DNA sequences mimicking defibrotide, a nucleic acid of genomic origin. We validated the remarkable affinity of CG51 for CatG with a K_d_ in the micromolar range, showing that the selection had effectively led to a pool of efficient binders.

Remarkably, the sequence analysis of the selected binders did not disclose a defined consensus motif, and well established algorithms used to evaluate folding of nucleic acids indicated that the identified aptamers do not tend to fold into stable and defined three-dimensional conformations. Due to the highly charged character of CatG, a reasonable hypothesis for the lack of a defined common consensus sequence is that the recognition process was essentially based upon the optimization of ionic interactions with DNA. Thus, through the selection protocol, we likely picked out molecules that, to better fit onto the accessible protein surface without limiting constraints, do not tend to fold into stable structures. Were this hypothesis true, high flexibility and optimal sequence length would represent the main factors responsible for the tight binding of some oligonucleotides to the positively charged protein.

Interestingly, CatG binders sequences were enriched in imperfect TG repeats. The analysis of binding and catalytic inhibition by alternating TG oligomers confirmed that these motifs may be considered excellent CatG binders and inhibitors. To confirm the hypothesis of a peculiar selection mechanism based on specific repeat motifs, the affinity of selected CatG binders was compared to that exhibited by alternating synthetic oligonucleotides. The dissociation constants for the oligos (dA-dC)_30_, (dT-dG)_30_ and for CG51-complementary sequence (cCG51) were all comparable and close to the values found for the oligos identified by Selex. Since all of the above compounds share a poor tendency to fold, the selection was indeed driven by the ability of the DNA molecule to stick onto the protein surface in an extended and flexible linear form. In the case of CatG, the sequence context plays a role exactly opposite to that generally played in producing selection of well defined and structured aptamers.

Notably, the sequences derived from the selection process mimics the abundance of TG repeats in genomic DNA and defibrotide, well known CatG inhibitors [21, 27]. TG rich sequences were found more frequently than AC steps in the selected aptamers. To account for this, it is reported that dG and dT nucleotides have faster coupling times during the random pool synthesis [41], therefore, sequences abundant in dA-dC are possibly under-represented in the starting pool.

The above considerations help explain the poorer CatG-binding properties of defibrotide. In fact, although this compound consists of a random mixture of ssDNA molecules of natural origin, they however largely undergo intra- as well as intermolecular folding (pairing) equilibria [25], thus producing an effective competition with the process of CatG binding.

A further indication for the requirement of an unfolded structure comes from the data regarding pE35, an aptamer of comparable length of GC51 but with a defined structure [38], that interacts poorly with CatG. A shorter but folded aptamer (THR) with high affinity for the serine protease thrombin was also evaluated for binding, exhibiting a poor affinity as well.

The need for proper DNA length to optimize the binding process has also been evidenced. Chromatography data proved that both linearity and length are essential for binding: molecules which are 60–80 nucleotides long are tight binders of CatG, while shorter or longer sequences showed poorer affinity. These data are in apparent contrast with SPR results, that evidenced a higher affinity for longer oligos in the series TG and AC. Using this technique the affinity appears to be almost linearly dependent upon the length of the tested oligos. To explain this we should consider that in SPR experiments one DNA chain can bind only one protein molecule since the enzyme is covalently attached onto the chip surface at low density. On the contrary, the Selex protocols, as well as the catalytic inhibition experiments, are based on incubations of protein and DNA in solution with the possible formation of different types of complex. Such behaviour has been previously reported for complex formation between CatG and glycosamminoglycans [16], negatively charged polyelectrolytes exhibiting an extended conformation similar to that shown by DNA-CatG binders.

Finally, the binding of tested oligonucleotide sequences to CatG induces a modulation in the catalytic properties of the enzyme, since CG51 and alternating oligos of the same size are efficient binders and inhibitors. Alternating oligopolymers of different length are also good inhibitors of CatG: indeed, the enzymatic properties of the protein are strictly dependent on the accessibility and conformation of its active site, and different binding mode(s) are likely to affect the catalytic response of the complex to different extents.

In conclusion, our selection allowed to identify the sequences of DNA binders of CatG from a combinatorial pool of oligodeoxyribonucleotides mimicking the molecular diversity of defibrotide, a known enzyme inhibitors of natural origin. More than one order of magnitude in affinity was gained relative to the natural product; it is foreseeable that subjecting CatG aptamers to a further Selex protocol may improve the affinity to submicromolar levels. The analysis of the selected binders unveiled a new paradigm for the recognition of a specific target by DNA sequences which, instead of being based upon a precise nucleic acid folding, rests on an extended, flexible binder conformation for maximal interaction with the target protein surface.

## 4. Experimental Section

### 4.1 Materials

Cathepsin G was purchased from Europa Bioproducts and dissolved before use in 50 mM sodium acetate (pH 5.5) and 150 mM NaCl. Defibrotide, a product derived from porcine double stranded DNA that is treated to produce a family of ssDNA molecules, was produced by Gentium SpA. All oligodeoxyribonucleotides were obtained from Eurogentec Bel SA (Belgium) and purified by PAGE before use.

The single stranded DNA (ssDNA) random pool used in the selection against cathepsin G is a 96-mer with a random region of 60 nucleotides flanked by two constant regions for the primers annealing. The estimated complexity of the pool was of 10^16^ molecules. Its sequence is: 5’-CGT ACG GAA TTC GCT AGC (N)_60_ GGA TCC GAG CTC CAC GTG-3’, with the underlined region referring to the EcoRI restriction site. The sequences of the PCR primers are respectively 5’-CGT ACG GAA TTC GCT AGC-3’ (*UP primer*) and 5’-CAC GTC GAG CTC GGA TCC-3’( *DOWN primer*). The latter primer has a biotin at its 5’ end.

Sequences of CatG binders and other oligos used as controls are:

CAACGTGTGATATGTGGGTATACGCTTGGGTGTTACGCTGAGCACAGAGGGTATTCGTGT (*CG51*), ACACGAATACCCTCTGTGCTCAGCGTAACACCCAAGCGTATACCCACATATCACACGTTG (*cCG51*), AATTCGCTAGCTGGAGCTTGGATTGATGTGGTGTGTGAGTGCGGTGCCCGGATCC (*PE35*), GGTTGGTGTGGTTGG’ (*THR*).

The sequences of the primers used for sequencing are ACG CCA AGC TTG CAT (*ELEA57*) and GGGTTTTCCCAGTCACGA (*ELES357*).

### 4.2 Selection of DNA-CatG binders

For the first Selex cycle 20 nmoles of the ssDNA random pool were large scale amplified by PCR in a mix (12.5 mL) containing amplification buffer 10x (1.25 mL), dNTPs (1.25 mM each, 2 mL), *Taq* polymerase (100 units, Pharmacia Amersham Biotech) and 25 nmoles of both UP primer and DOWN primer. Eight PCR cycles were performed in water baths with this temperature scheme: 4 minutes 94 °C, 7 minutes 50 °C and 7 minutes 72 °C. The pool was then precipitated in ethanol/acetate, resuspended in TE (300 μL) and purified through a G-50 column.

To generate ssDNA after each PCR we used an alkaline denaturation protocol. Briefly the biotinylated DNA, resuspended in SBB buffer (Streptavidin Binding Buffer: NaCl 50 mM, Tris/HCl 100 mM, EDTA 10 mM) was loaded in a chromatography column filled with streptavidin Sepharose resin (Pierce). After 30 minutes incubation, the unbound dsDNA was washed away with SBB buffer while the non-biotinylated strand was collected by washing the column with NaOH 0.15 N. After precipitation the ssDNA concentration was measured by UV spectroscopy and used for the Selex cycles.

The random pool obtained after large scale PCR amplification and alkaline elution from the streptavidin resin was radioactive labelled at 5’ end with [γ-^32^P]ATP (NEN Life Science) and T4 polynucleotide kinase (Amersham). The labelled ssDNA was then denatured at high temperature, renatured and incubated with Cathepsin G in Incubation Buffer (IB buffer: 30 mM Tris HCl pH7.5, 150 mM NaCl, 5 mM KCl and 5 mM MgCl_2_). NaCl concentration was chosen to mimic the physiological condition. The incubation was performed for 90 minutes in ice. The sample was then loaded in a mini-column (Biorad) filled with Sepharose SP resin (Amersham Pharmacia Biotech) previously swollen and equilibrated in buffer IB. The ssDNA/protein solution was incubated with the resin for 30 minutes at 4 °C. The unbound oligonucleotides were washed away with buffer IB, while the Cathepsin bound sequences were eluted from the column with a high ionic strength buffer (EB buffer: 0.8 M NaCl and 50 mM Tris pH 7.8). The eluted fractions were counted and the yield of the Selex cycle was expressed as a percentage of the total radioactivity. The flow through and the first EB washes, which contain the largest amount of bound ssDNA, were collected, amplified by PCR (90°C for 1 min, 50°C for 1 min, 72 °C for 1 min) and rendered single stranded as described above. The number of PCR cycles never exceeded 20. Double stranded DNA after each PCR was gel purified through a native 8% PAGE (Tris-borate 89 mM EDTA 2 mM).

In order to avoid unspecific binding of ssDNA molecules to the SP resin, two precolumn cycles were performed after cycle 6 and 8: the ssDNA pool (without CatG) was loaded in a Sepharose SP column and washed with buffer IB. The DNA molecules in the first fractions, representing non-matrix binders, were collected, precipitated and PCR amplified using *Taq* polymerase (Amersham) at a concentration of 0.3–0.5 U/50 μl in the buffer provided by the supplier, and used in subsequent selection cycles.

### 4.3 Cloning and sequencing

Before the insertion in the plasmid vector for cloning, the DNA was subjected to a *polishing reaction* in order to get blunt ends: an aliquot of the normal PCR reaction, performed with non-biotinylated Down primer, was incubated with *Pfu Turbo polymerase* (2.5 U/μL, Stratagene), in the suggested buffer at 72°C for 30 minutes. The amplified dsDNA was cleaved with 2.5 units of EcoRI at the 5′ end. The plasmid vector pUC19 (Amersham) was treated with EcoRI and SmaI that cleaves giving blunt ends. After precipitation 3 pmols of dsDNA and 0.6 pmols of pUC19 were reacted with T4 ligase in the suggested buffer. The plasmid was then inoculated in *E.coli* competent cells (SURE strain by Stratagene) by the electroporation method using *E.coli pulser* (Biorad) and plated in solid LB media in the presence of Ampicillin (45 μg/mL), X-Gal (40 μg/mL) and IPTG (0.5 mM) for the blue/white screening. Fifty white different colonies were picked, grown in liquid LB broth (Becton Dickinson) and harvested. The plasmids were purified by alkaline lysis and their quality was tested every time by agarose gel electrophoresis. The sequence of the aptamers was determined using T7 Sequenase (Amersham) and [α-^33^P]dATP (Perkin Elmer Life Sciences); the primers EleA457 and EleS357 were used for sense and antisense reading respectively.

### 4.4 K_d_ determination

The dissociation constant of each oligonucleotide was determined by chromatography in analogy with the selection protocol. The labelled oligonucleotides (600 pmoles) were incubated 30’ in ice with the protein and then loaded onto a mini-chromatography column filled with Sepharose SP (300 μL, Amersham Pharmacia Biotech) and incubated for further 30’. The column was washed with 15 volumes of buffer IB, then after one further hour of incubation with buffer EB, it was washed with six volumes of this buffer. Each volume was collected and counted. Three independent experiments were performed for each test sequence using a constant amount of the oligonucleotide and variable amount of Cat G (5, 10 and 15 μg). K_d_s were calculated approximating a first order reaction.

### 4.5 Surface Plasmon Resonance (SPR) experiments

CatG, from human neutrophils, dissolved in HBS EP buffer, pH 7.40 was immobilized on the surface of a CM 5 research grade sensor chip flow cell, according to the procedure suggested by Biacore and using the Biacore amine coupling kit (Biacore). A blank flow cell was prepared using all the above reagents but CatG. The amount of CatG immobilized on the surface of the flow cell was 5178.91 ± 129.63 RU. Tested aptamers were dissolved in Tris-HCl buffer, pH 7.50 (30 mM), NaCl (150 mM), KCl (5 mM) and MgCl_2_ (5 mM) and injected over the CatG surface or the blank surface. Experiments at variable (molar or weight) oligonucleotide concentration were performed. All the above experiments were run at 25° C, using as running buffer the Biacore HBS EP Buffer, pH 7.4. The CatG surface was regenerated by two injections of 2 M NaCl. The blank sensorgram was subtracted from each sample sensorgram and the binding response evaluated. The binding responses, generated in the third set of experiments, were plotted as a function of the Log concentration (nM) to get concentration-effect curves to find out the relative potencies of aptamers in binding CatG.

### 4.6 Enzymatic assays

The inhibition of the catalytic activity of CatG was evaluated following the extent of hydrolysis of a proper chromogenic substrate induced by the proteinase in the presence/absence of test oligonucleotides. A solution of 600 nM CatG was incubated for 30 minutes at room temperature with increasing concentrations of each oligonucleotide in pH 7.4 phosphate buffer (50 mM), PEG (0.1%). The experiments in the presence of albumin were performed using 600 nM of human albumin. The substrate Suc-Ala-Ala-Pro-Phe-pNA (Calbiochem) was then added to a final concentration of 350 μM. The hydrolysis of p-nitroaniline was followed as a function of time recording the absorbance at 410 nm with a Perkin Elmer Lambda 20 spectrophotometer. The initial rate of substrate hydrolysis was evaluated from the slope of a plot of absorption at 410 nm vs. time. The ratio of the initial rate in the presence/absence of the inhibitor, (v_i_/v_0_), has been plotted as a function of DNA concentration, and the oligonucleotide concentration which induces a 50% of the maximal reduction of enzyme activity has been graphically evaluated. Measurements performed in the presence of increasing concentrations of substrate allowed us to evaluate a K_m_ = 7.1 mM well in accordance with literature data [42].

## Figures and Tables

**Table 1.** Sequences of the selected DNA-CatG binders

Clone	DNA sequence
**CG1**	GGGTGGCCCCCTAGTCGCGCACTGGAAGCGGTAGTGTCGTGAGATTCGTATCTGGGGTAT
**CG3**	CAACGAGTCAGGGCGTGATTGGTGAAGATGTGTGGTTTGGCCAGAAAGGGCGATGGTGGA
**CG11**	AGAGCTGAGACGGACATGCTGCCCATGGAGACTGTTCGAGAGGGTGAGCGGGAGTGGG
**CG16**	ACCCCTAGGTCAGCACGTAGTGTAGGGCGATGTGTTCATGGCGGGAATGTGAGTTGTGGG
**CG20**	GGGCGGCTCGCGTTGTGGAACATTCGTGGTGCCAATGCGTACCAGGGATTGCCTCCTGT
**CG25**	GGGCGATTGGCGAATGCAAGGGTAAGGTTGGGCGATTGATGTGCACGTAGCGCAGAGCAT
**CG28**	XXGGAACGTGGTAGGTGTGTCTGCTGTGTGTGGCTCGGGCAGGTTGTCAGGGTGTTT
**CG32**	GGGCATAGGGCGTCGTAGCCTGAAGGTGTGATTCGTGCGTTAGATGGGGGGCAGTCTGC
**CG43**	CAACGTGTGATATGTGGGTATACGCTTGGGTGTTACGCTGAGCACAGAGGGTATTCGTGT
**CG45**	GGCGGGCGGTATGGGCTGCAGGATATGCAGGGGCGCAGAGGACAGTCTGGCCATGTACTA
**CG49**	GGCCTGGGTGATGTACTATGTATGCGTCGTGGTGGCTGGTAAAGGGGGTCTGCTATGGGT
**CG51**	CAACGTGTGATATGTGGGTATACGCTTGGGTGTTACGCTGAGCACAGAGGGTATTCGTGT
**CG2**	CCACGGACGCTGTGAGCGGCCAACGGATGGGAATCACGATCTGGCCCGAACCACATACCG
**CG31**	TCACACTAGGGCACTTGCTAAGTAGCTATGTAACTCGATCATACTTATTAGGCTTG
**CG23**	AATCGATGGACACTTCAACGCAACTTGACATGGCGGTACGTGGACTCTTGTGGCGACAGTT
**CG34**	AACCCGTGTGATAAGGATATGGTGACTTCGTGGCACAGCGTCGACGGACTGCCCATTCCA
**CG40**	GGCAGGGACGTTCCCAGGAATGCGGCACAGGCAGACAGCTCCCGACGAGTACCAGGGTG
**CG48**	AGXGGGCAGCAGCACACCACACATGTACGTGGGGGATTGCATTGTGTACTTAGACGGTAT
**CG39**	CGGTGGAGAGGTCGCAATGACACGGTTGACGATAGGCCCCTTGCTAACATCGGTTGGTG
